# Supplementary material for: The effect of MurM and a branched cell wall structure on penicillin resistance in Streptococcus pneumoniae
Source: J Bacteriol. 2025 Oct 8;207(11):e00141-25. doi: 10.1128/jb.00141-25 (PMC12632259; doi:10.1128/jb.00141-25)
Supplement: Supplemental figures and tables — Figures S1 to S12 and Tables S1 to S5. [file jb.00141-25-s0001.docx]

Supplementary Material

# The effect of MurM and a branched cell wall structure on penicillin resistance in *Streptococcus pneumoniae*

Ragnhild Sødal Gjennestad^1^, Maria Victoria Heggenhougen^1^, Anja Ruud Winther^1^, Johanne Moldstad^1^, Vegard Eldholm^2^, Morten Kjos^1^, Leiv Sigve Håvarstein^1^, Daniel Straume^1^

^1^Faculty of Chemistry, Biotechnology and Food Science, Norwegian University of Life Sciences, Ås, Norway

^2^Department of Bacteriology, Norwegian Institute of Public Health, Oslo, Norway

# Supplementary figures


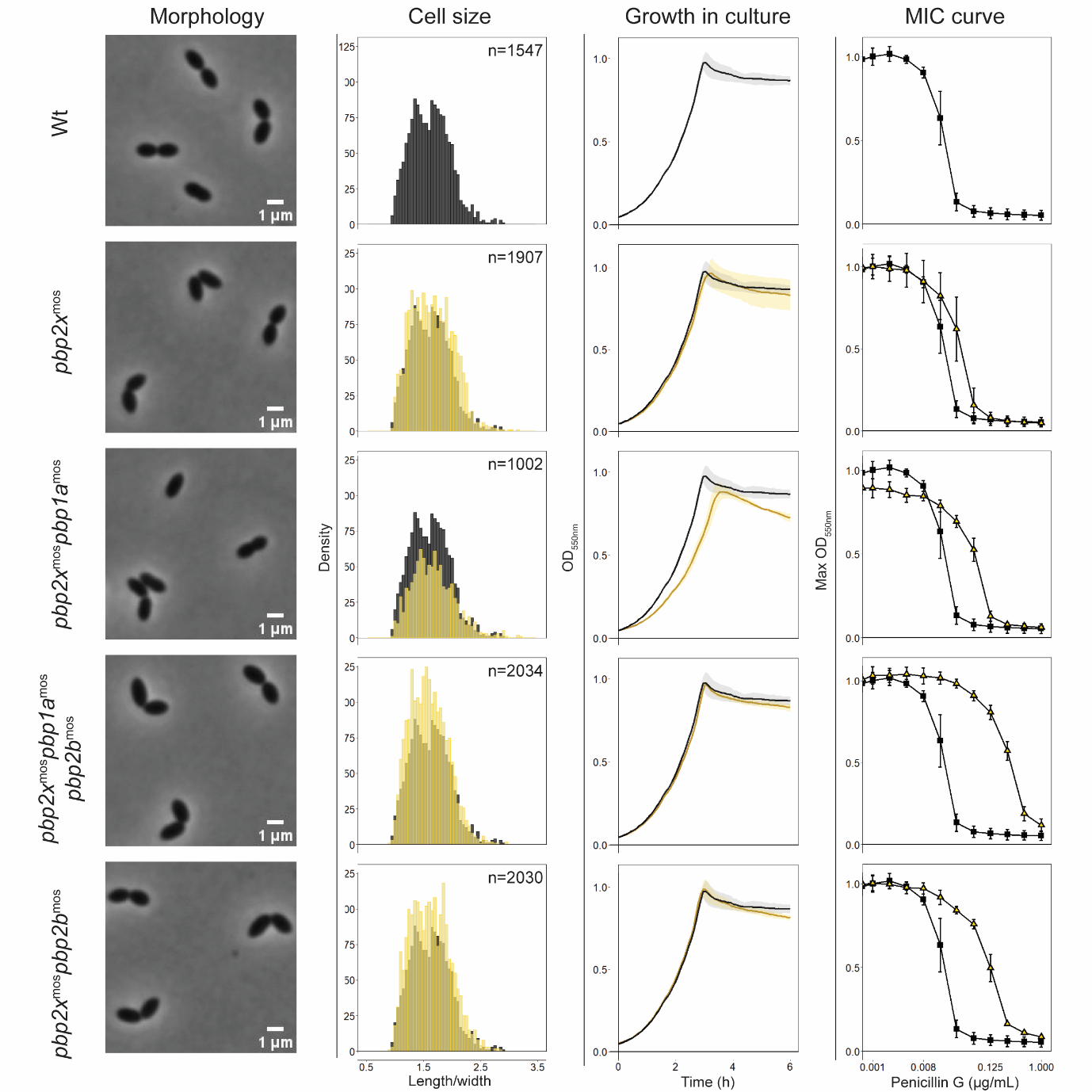


Figure S1: Phenotypical characteristics of Wt and mutants with different combinations of low-affinity PBPs. Cultures were diluted to an OD_550nm_ of 0.05 and grown to an OD_550nm_ of 0.4 for phase contrast microscopy. Images display a representative view of the different mutants. Cell size distribution was calculated based on length/witdh ratios, with meassurements performed by the ImageJ and MicrobeJ plug-in. The numbers of cells included in the analysis are indicated. Bacterial growth (OD_550nm_) in liquid culture was measured continuously every 5 minutes for 16 hours (only the first 6 h are displayed). The MIC curves display maximum OD_550nm_ at each PenG concentration (a two-fold dilution series starting at 1 µg/mL) with the MIC_50_ value (Table 1) determined by the PenG concentration that inhibited ≥50% of the maximum OD_550nm_. Wt measurements are shown in black, and the different low-affinity *pbp* mutants (specified to the left in the figure) are show in yellow. Standard deviation was calculated from three biological replicates.


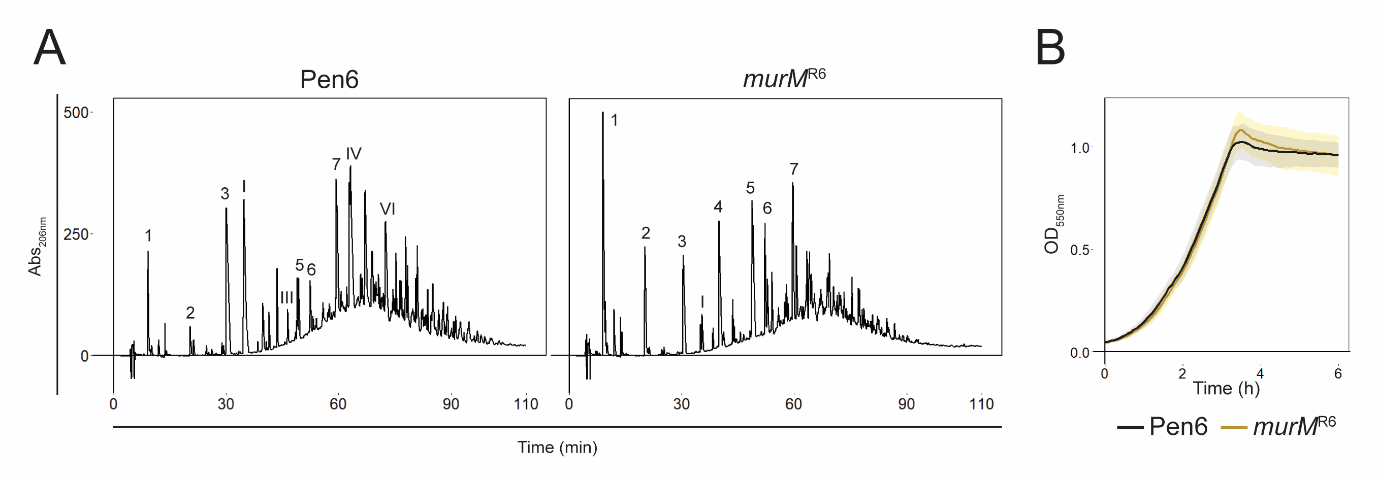


Figure S2: Stem peptide analysis of peptidoglycan from Pen6 and a mutant in which the highly mutated *murM^Pen6^* has been replaced with *murM^R6^*. (A) Cell wall of exponentially growing cultures (OD_550nm_=0.4-0.5) was isolated, treated with LytA and the stem peptides were separated using C18 reverse phase HPLC. The stem petides of the numbered peaks are illustrated in Figure S3. The replacement of *murM* resulted in a shift from consisting of mostly branched stem peptides to a more Wt-like cell wall consistent with previous research (1). (B) Growth curves of Pen6 and Pen6 *murM*^R6^. Replacement of *murM^Pen6^* with *MurM^R6^* did not influence growth.


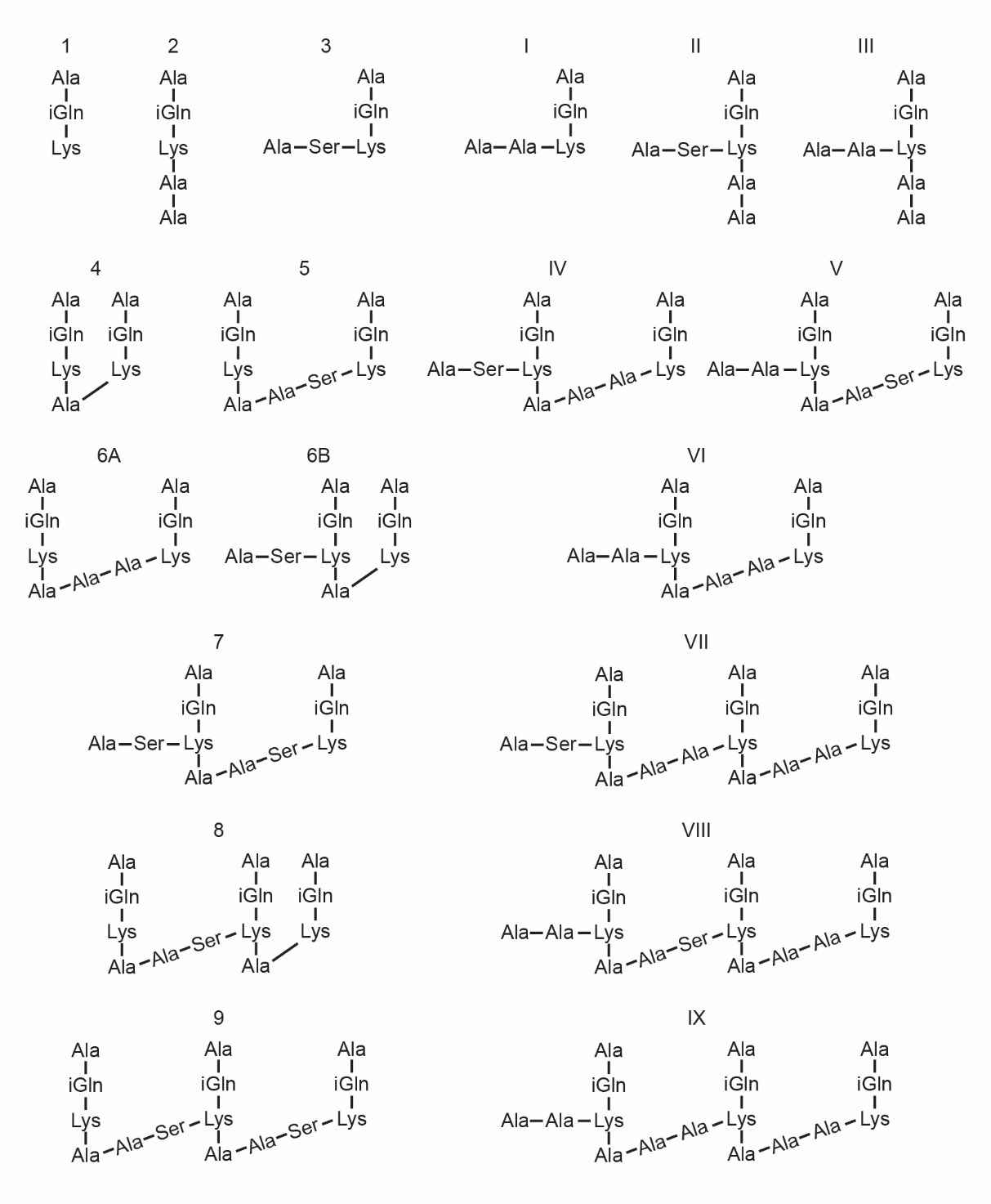


Figure S3: Structures of pneumococcal cell wall stem peptides. The stem peptides structures have been published previously (2-4).


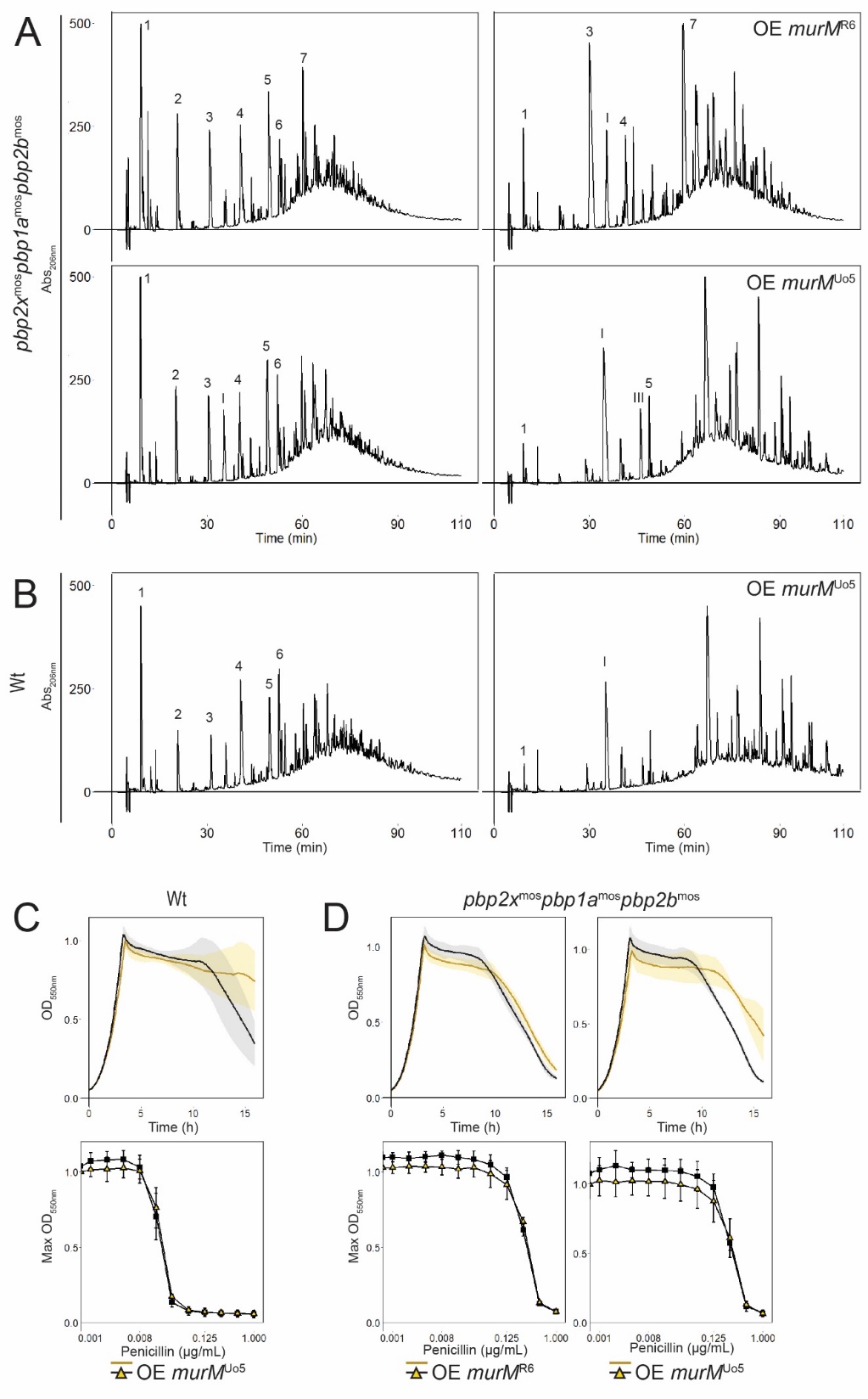


Figure S4: Phenotypical characteristics of *murM* overexpression in Wt and a low-affnity PBP background. *murM* from either R6 or Uo5 (as indicated in the figure) was placed under control of an inducible promoter (P*_comX_* using the ComRS system (5)) in Wt (B, C) or the *pbp2x*^mos^*pbp1a*^mos^*pbp2b*^mos^ mutant (A, D). (A, B) Cultures were induced with ComS (0.2 µM) at OD_550nm_ = 0.05 and cell wall of exponentially growing cultures (OD_550nm_ = 0.4-0.5) was isolated, treated with LytA and the stem peptides were separated using C18 reverse phase HPLC. Stem peptide sturctures of the indicated peaks are illutrated in Figure S3. Overexpression (OE) of *murM* resulted in a clear shift from a linear to a more branched cell wall structure. (C, D) Cultures were diluted to an OD_550nm_ of 0.05, inducer (0.2 µM ComS) was added, and bacterial growth (OD_550nm_) was measured continuously every 5 minutes for 16 hours. The MIC curves display maximum OD_550nm_ at each PenG concentration (a two-fold dilution series starting at 1µg/mL). The black lines and points represent control measurements (without inducer) and the yellow lines and points represent *murM* overexpression. Standard deviation was calculated from three biological replicates. Overexpression of *murM* had no effect on MIC_50_ or growth rate but showed delayed autolysis.


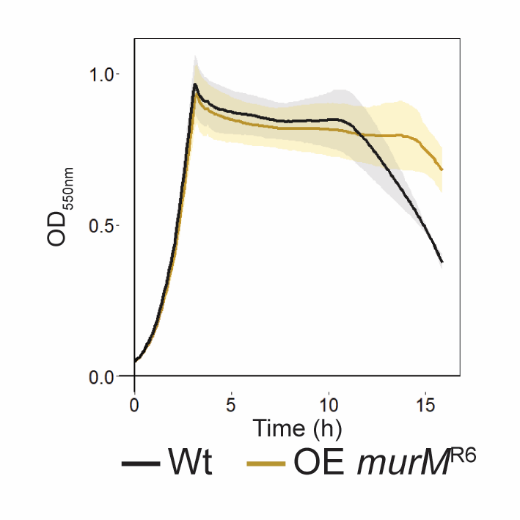


Figure S5: Growth during *murM* overexpression in Wt. *murM*^R6^ was overexpressed (OE) in Wt using the ComRS system. Cultures were diluted to an OD_550nm_ of 0.05, inducer (0.2 µM ComS) was added, and bacterial growth (OD_550nm_) was measured continuously every 5 minutes for 16 hours. Error bars represent the standard deviation calculated from three biological replicates. Overexpression of *murM* had no effect on growth rate but showed delayed autolysis.


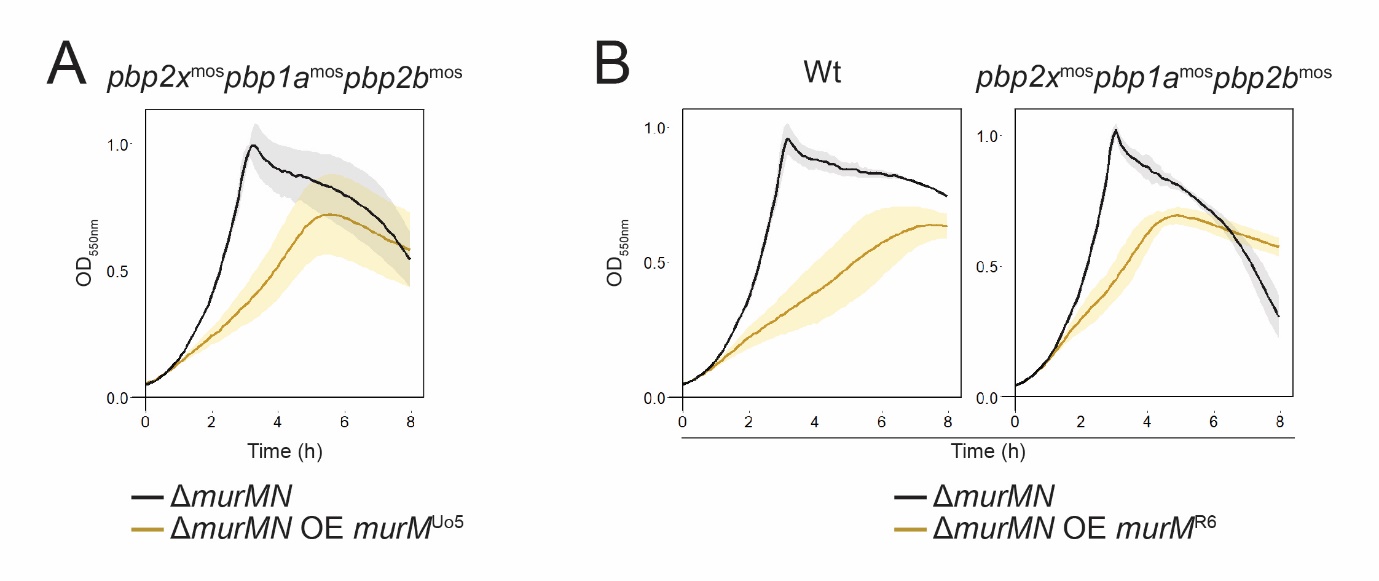


Figure S6: Impact of *murM* overexpression in Δ*murMN* mutants. *murM^Uo5^* (A) and *murM^R6^* (B) was overexpressed (OE) in Δ*murMN* mutants of Wt and *pbp2x*^mos^*pbp1a*^mos^*pbp2b*^mos^ cells using the ComRS system. Cultures were diluted to an OD_550nm_ of 0.05, inducer (0.2 µM ComS) was added and bacterial growth (OD_550nm_) was measured continuously every 5 minutes for 16 hours (only the first 8h are displayed). Standard deviation was calculated from three biological replicates. A toxic effect of *murM* overexpression was observed when *murN* was deleted.


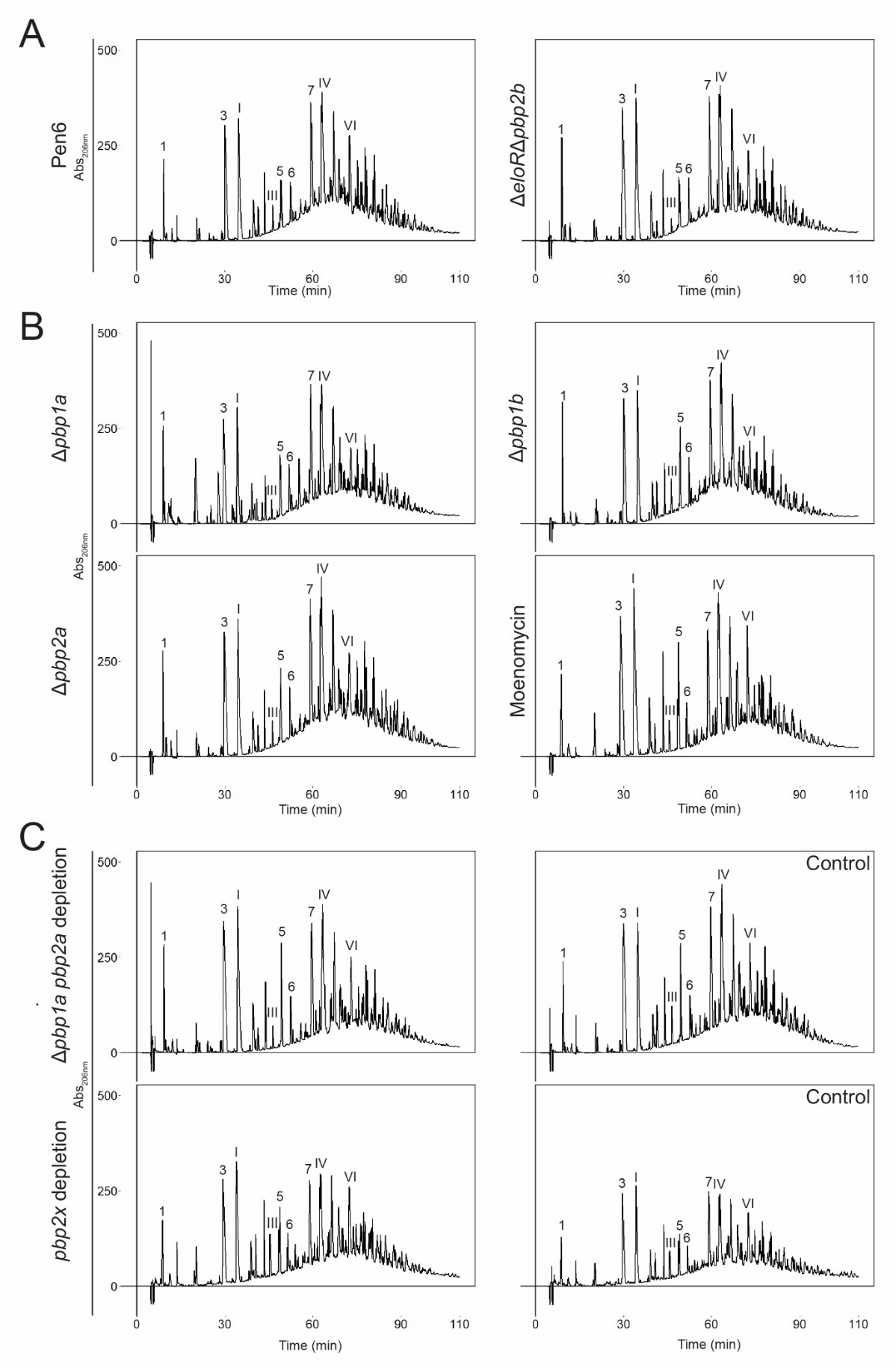


Figure S7: Cell wall stem peptide composition of Pen6 *pbp* mutants. Cell wall was isolated from cells at OD_550nm_ = 0.4-0.5, treated with LytA and the stem peptides were separated using C18 reverse phase HPLC. Stem peptide structures of the indicated peaks are illutrated in Figure S3. The area under the peaks were quantified and percentage of each component are listed in Table S3. (A) The parental strain Pen6 and a double Δ*eloRΔpbp2b* mutant. No major differences were observed in the mutant. (B) Stem peptide profiles of single Class A *pbp*s mutants and moenomycin (5 µg/mL) treated cells. The moenomycin specifically targets the Class A PBPs and cell wall was isolated from growth inhibited cells. Knockout or inhibition of class A PBPs had little influence on the cell wall composition. (C) Stem peptide profiles of cells depleted for *pbp2a* in a Δ*pbp1a* background and cells depleted of *pbp2x*. The *pbp*-depleted cells were harvested at the point where cells grew poorly from lack of the PBP, but were still viable. No evident changes to the stem peptide composition were observed.


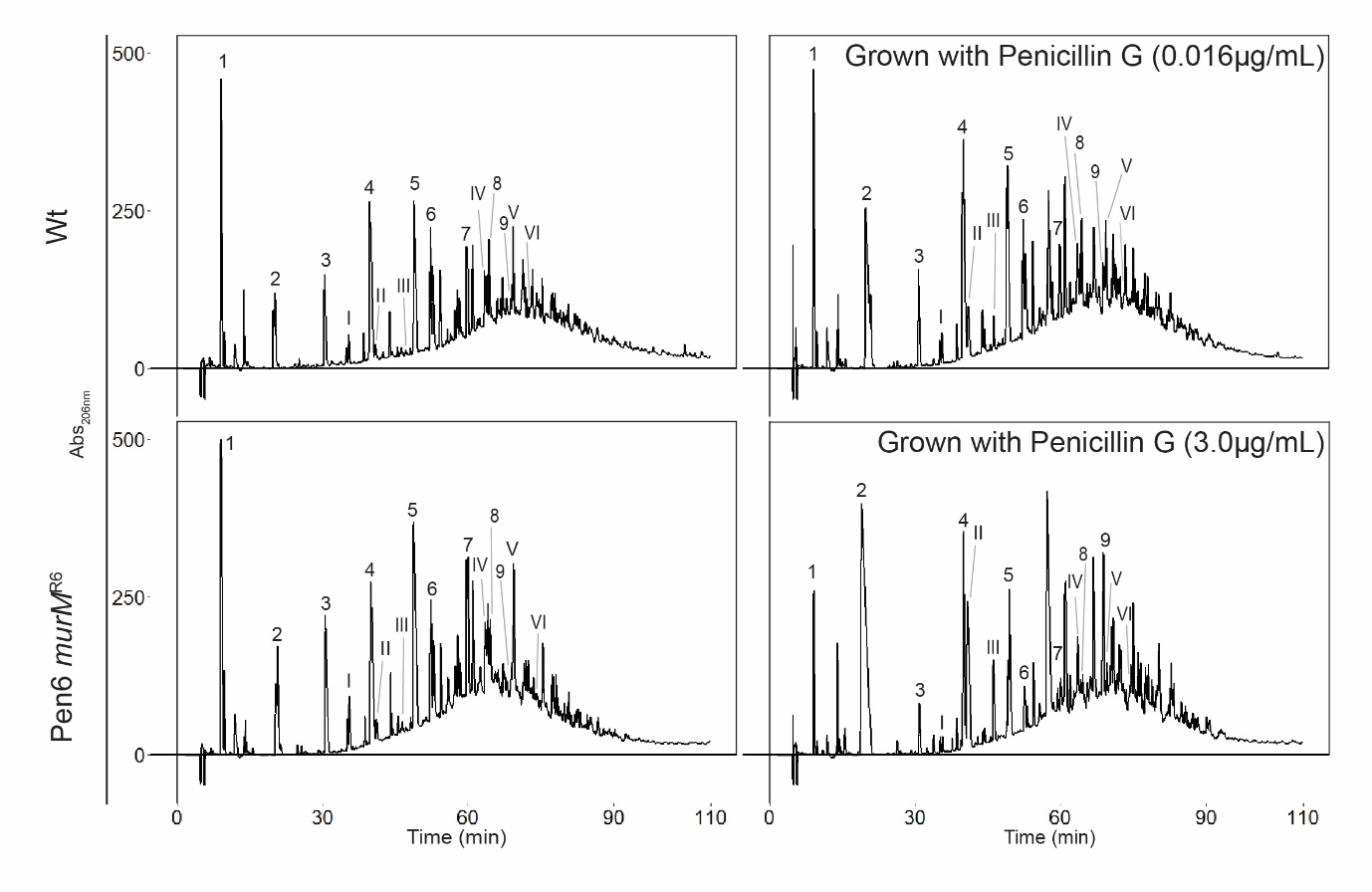


Figure S8: Changes to the cell wall stem peptide composition during penicillin exposure. Stem peptide profile of Wt and a Pen6 *murM*^R6^ mutant when grown with subinhibitory concentrations of PenG (indicated) compared to non-treated controls. PenG was added to the growing culture at OD_550nm_ = 0.05 and cell wall was isolated when the cultures reached OD_550nm_ = 0.4-0.5. Stem peptide structures of the indicated peaks are illutrated in Figure S3. The area of the peaks of the stem peptides were quantified and percentage of each component are listed in Table 2. Upon PenG exposure, the peptidoglycan composition of both strains displayed a percentage incresae in monomeres as well as linear peptides relative to branched peptides.


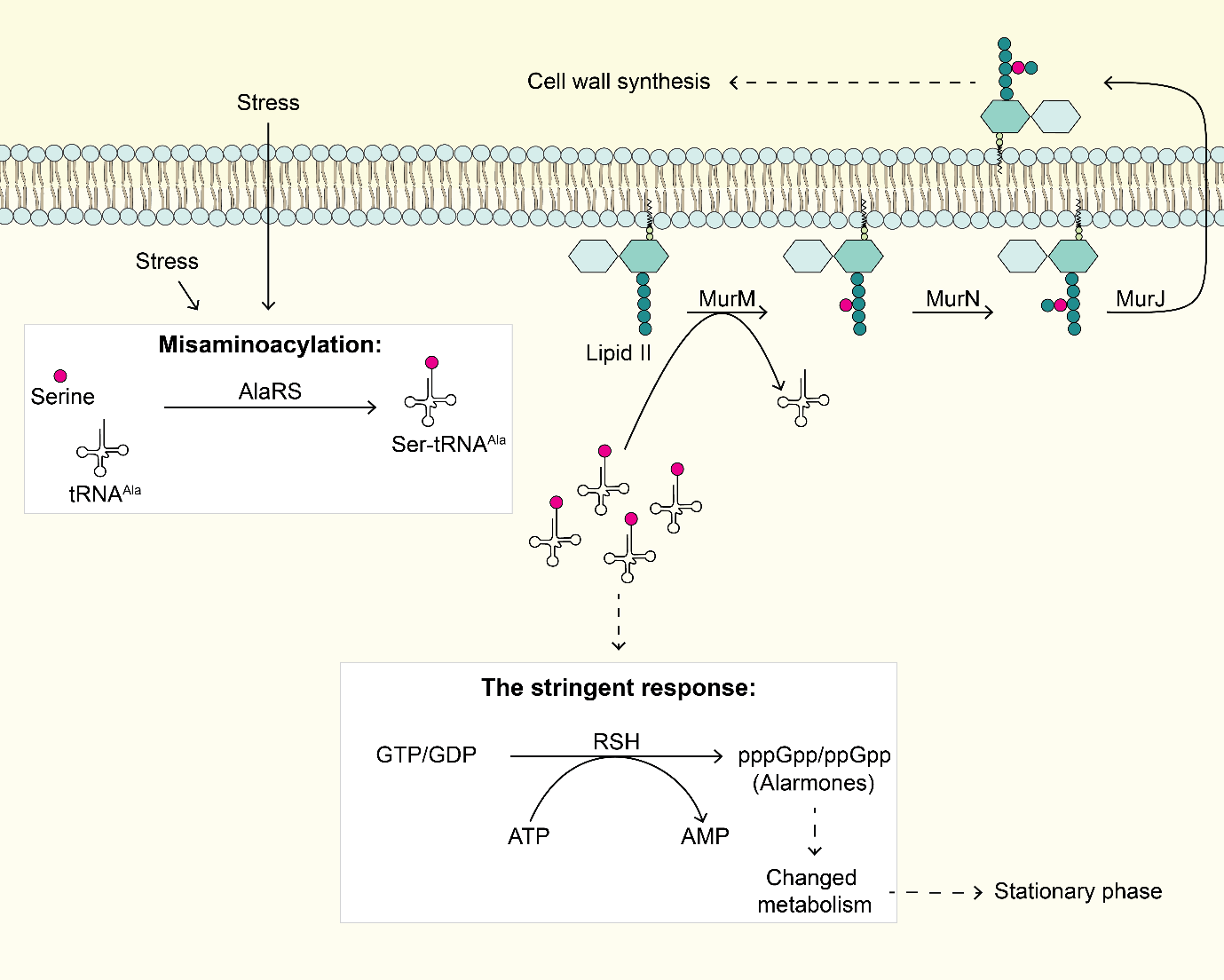
 Figure S9: The interpaly between the stringent response and MurM in pneumococci. Alanyl-tRNA^Ala^ synthetase (AlaRS) recognizes alanine and synthesizes Ala- tRNA^Ala^ but it can also mistakenly recognize serine, leading to misaminoacylated Ser- tRNA^Ala^. AlaRS has editing activity and can edit the misaminoacylated tRNAs, but the enzyme is error prone, leading to mismatched tRNAs. Stress (acidic stress was previously tested) leads to a higher number of misaminoacylated Ser-tRNA^Ala^, which could lead to translation errors. A paper from Aggarwal et al. (2021) found that MurM is likely to work as a buffer for missaminoacylated Ser- tRNA^Ala^ by favouring incorporation of these serine amino acids into the cell wall. Deletion of *murMN* under acidic stress led to accumulation of Ser- tRNA^Ala^, activating the stringent response pathway. Activation of the stringent response triggers production of a large amount of alarmones (pppGpp or ppGpp) in the cells. In Gram positives, the alarmones are produced by the RSH protein (RelA/SpoT homolog) that has both synthesase and hydrolase activity. Increased alarmone levels leads to changes in the cell’s metabolism and coordinate the entry into stationary phase.


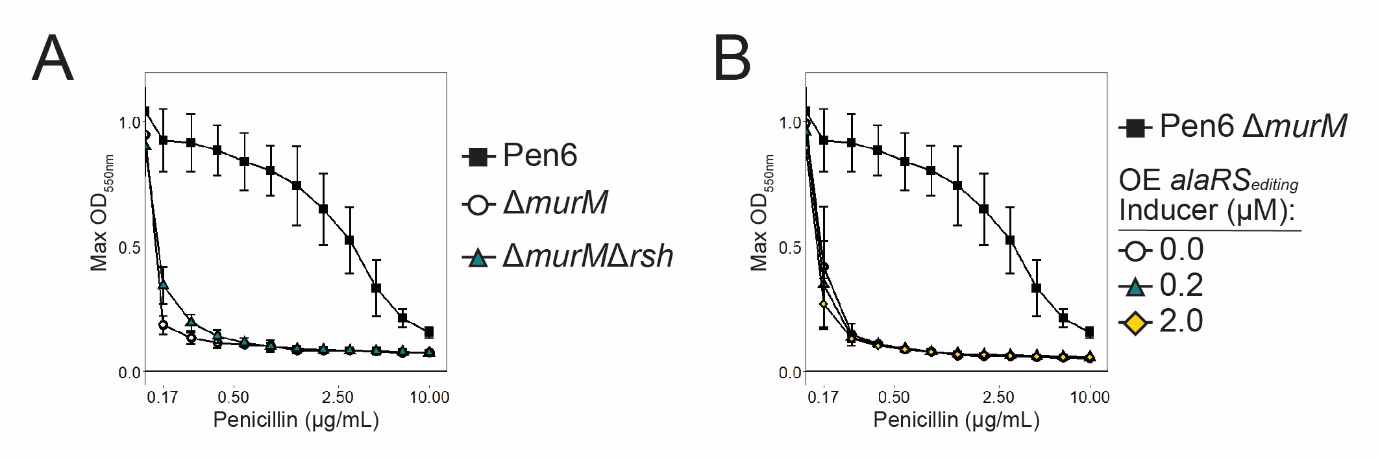


Figure S10: Inhibition of the stringent response in Δ*murM* mutants. Panel A shows the PenG MIC curves of Pen6 and its Δ*murM* and a double Δ*murM*Δ*rsh* mutant. Deletion of *rsh* in a Δ*murM* mutant did not revert the initial phenotype of the strain. (B) The editing domain of AlaRS (*alaRS_editing_*) was ectopically expressed using the ComRS system in a Pen6 Δ*murM* mutant. Overexpression (OE) of AlaRS_editing_ using 2 µM ComS inducer gave no change in MIC_50_ in the Δ*murM* mutant. Error bars represent standard deviation calculated from three biological replicates.


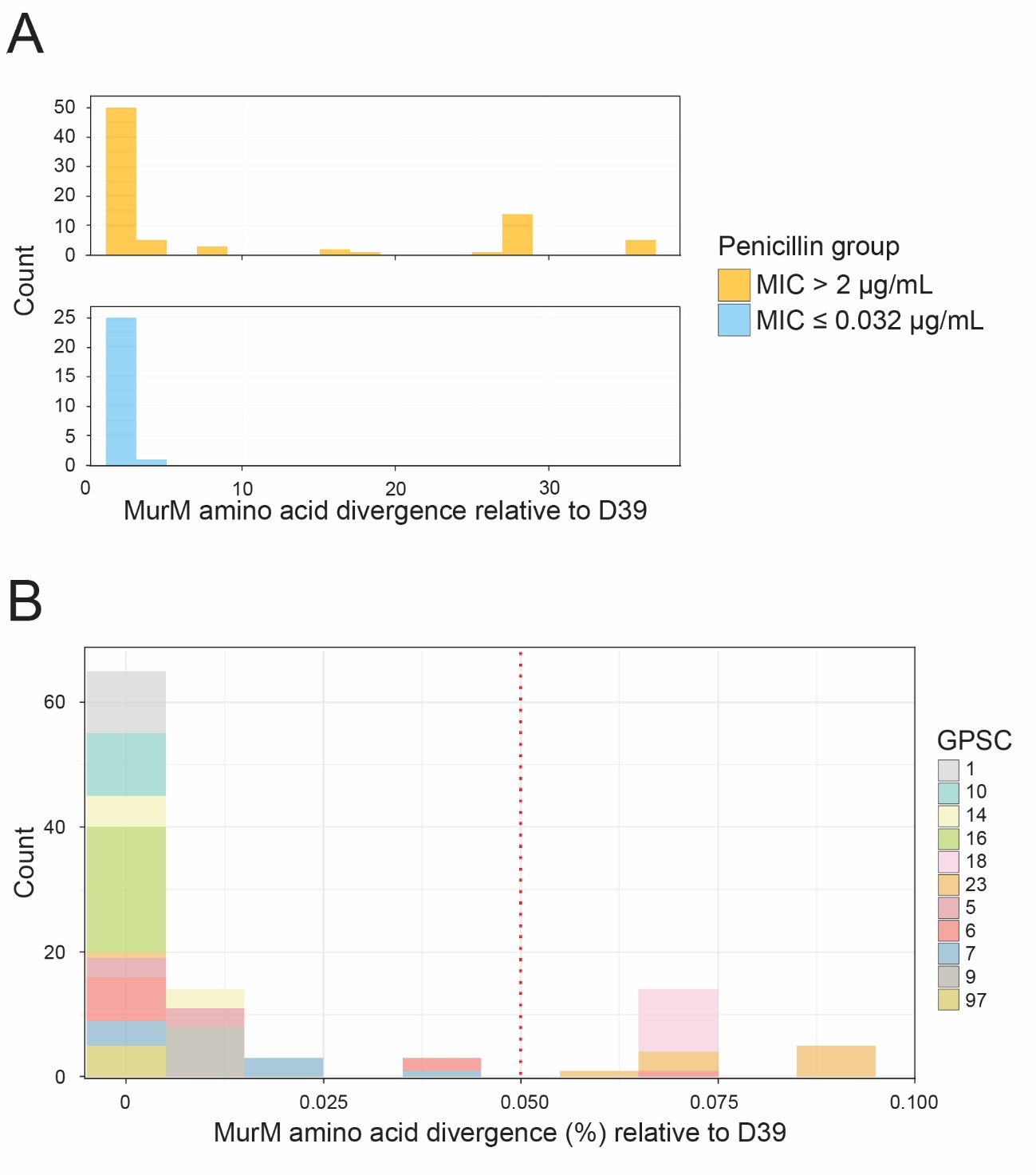


Figure S11: MurM divergence and PenG MICs. (A) Histogram of MurM divergence (amino acid differences) relative to the D39 reference, stratified by MIC groups (resistant isolates on top, susceptible bottom). (B) Histogram of MurM divergence (%) relative to the D39 reference, colored by GPSC type. The Wt used in this study (R6) is a non-encapsulated derivate of the strain D39 and have identical MurM sequences.


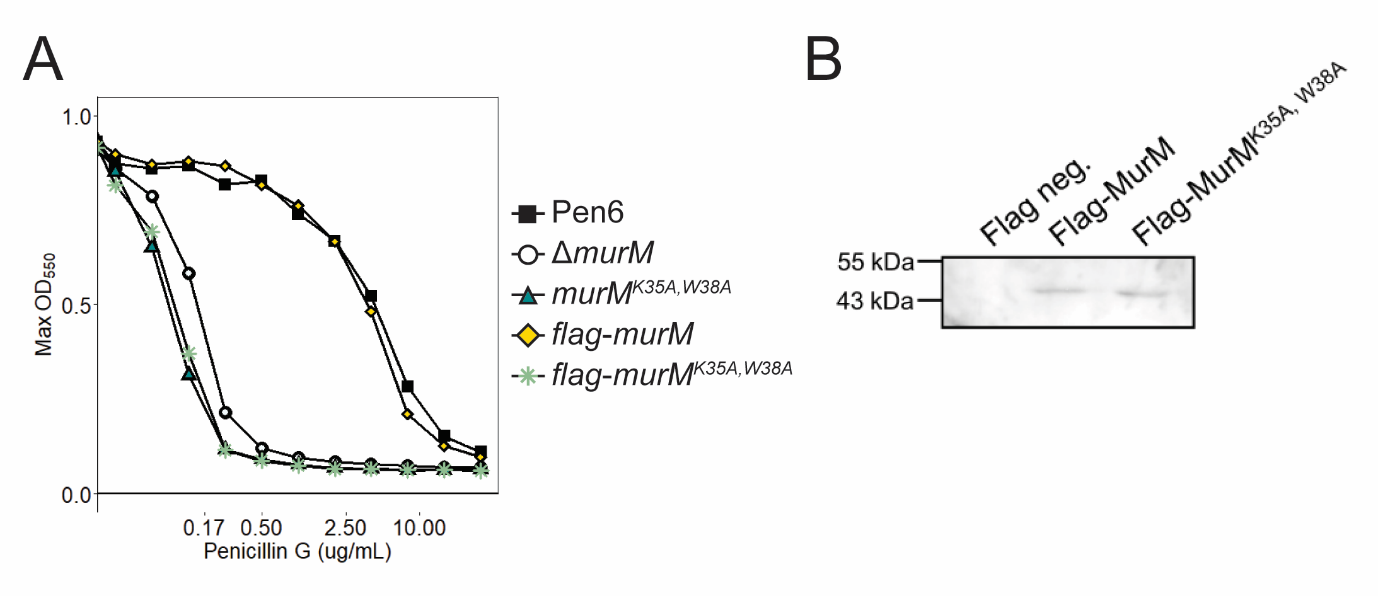


Figure S12: Resistance profile of a catalytically inactive MurM mutant. A. Curves display maximum OD_550nm_ obtained at each PenG concentration. Presence of a non-functional MurM (i.e., essential amino acid recidues for lipid II binding replaced) did not rescue the Δ*murM* phenotype in Pen6. B. Immunoblot detecting Flag-MurM and Flag-MurM^K35A,W38A^. Wt was used as Flag-tag negative control.

Supplementary Tables

Table S1: Generic differences between the *pbp2x*^mos^*pbp1a*^mos^*pbp2b*^mos^ tripple mutant (MH83) and the Wt (RH425)

| **Gene** | **Locus tag** | **DNA difference** | **Protein difference** |
| --- | --- | --- | --- |
| *pbp2x* | spr0304 | c.C822-C2231 🡪 *pbp2x*^Uo5^ T825-A2234 | p.Q281-T745 🡪 PBP2x^Uo5^ L284-K246 |
| *mraY* | spr0305 | c.12C🡪T  c.15C🡪T  c.120G🡪A | None  None  None |
| *pbp1a* | spr0329 | c.G1596-A1950 🡪 *pbp1a*^Uo5^ T1593-G1947 | p.D533-N643 🡪 PBP1a^Uo5^ E534-S644 |
| *recR* | spr1516 | Replaced by *recR*^Uo5^ | Replaced by RecR^Uo5^ |
| *pbp2b* | spr1517 | c.A1776-* 🡪 *pbp2b*^Uo5^ T1761-* | p.A596-Y681 🡪 PBP2b^Uo5^ S592-H677 |
| *dltA* | spr1982 | c.838C🡪T | p.280A🡪T |
|  |  |  |  |

* Indicates STOP codon

Table S2: Stem peptide composition of Wt and low affinity PBP mutants.

| **Peak** | **Peptide characterisitcs** | | **Wt** | ***pbp2x*^mos^** | ***pbp2x*^mos^*pbp1*a^mos^** | ***pbp2x*^mos^*pbp2b*^mos^** | ***pbp2x*^mos^*pbp1a*^mos^*pbp2b*^mos^** |
| --- | --- | --- | --- | --- | --- | --- | --- |
| 1 | Linear | Monomer | 17.1 | 16.9 | 18.6 | 19.3 | 18.3 |
| 2 | Linear | Monomer | 3.9 | 9.0 | 9.8 | 10.9 | 9.3 |
| 3 | Branched | Monomer | 7.0 | 9.1 | 5.5 | 10.6 | 14.2 |
| I | Branched | Monomer | 1.5 | 1.8 | 1.9 | 2.4 | 2.8 |
| 4 | Linear | Dimer | 20.9 | 16.4 | 28.7 | 10.3 | 10.2 |
| II | Branched | Monomer | 0.9 | 0.9 | 0.4 | 1.9 | 1.7 |
| III | Branched | Monomer | 0.4 | 0.2 | 0.2 | 0.7 | 0.4 |
| 5 | Branched | Dimer | 16.1 | 13.6 | 7.8 | 16.5 | 13.7 |
| 6A | Branched | Dimer | 7.4 | 6.6 | 9.1 | 5.8 | 4.9 |
| 6B | Branched | Dimer | 5.8 | 5.5 | 5.2 | 3.0 | 4.0 |
| 7 | Branched | Dimer | 4.0 | 8.9 | 4.1 | 10.2 | 10.8 |
| IV | Branched | Dimer | 4.3 | 5.7 | 4.7 | 2.9 | 4.7 |
| 8 | Branched | Trimer | 5.3 | 3.0 | 2.7 | 1.8 | 1.5 |
| 9 | Branched | Trimer | 1.0 | 1.0 | 0.8 | 1.3 | 1.6 |
| V | Branched | Dimer | 4.3 | 1.4 | 0.5 | 2.3 | 1.8 |
| **Total** | | | 100 | 100 | 100 | 100 | 100 |
| **Monomers (%)** | | | 30.8 | 38.0 | 36.4 | 45.8 | 46.8 |
| **>Monomers (%)** | | | 69.2 | 62.0 | 63.6 | 54.2 | 53.2 |
| **Linear peptides (%)** | | | 41.9 | 42.4 | 57.1 | 40.5 | 37.8 |
| **Branched peptides (%)** | | | 58.1 | 57.6 | 42.9 | 59.5 | 62.2 |
| **Ratio peptide 5/4** | | | 0.8 | 0.8 | 0.3 | 1.6 | 1.3 |
| **B/L peptides** | | | 1.4 | 1.4 | 0.8 | 1.5 | 1.6 |

|  | **Molecular** | **Peptide** | |  |  |  |  |  |  |  | **P*_comX-_pbp2a*. Δ*pbp2a*Δ*pbp1a*** | | **P*_comX-_pbp2x*.**  **Δ*pbp2x*** | |
| --- | --- | --- | --- | --- | --- | --- | --- | --- | --- | --- | --- | --- | --- | --- |
| **Peak** | **Weight^a^** | **characteristics** | | **Pen6** | **Δ*pbp1b*** | **Δ*pbp2a*** | **Δ*pbp1a*** | **Δ*eloR*** | **Δ*eloR*Δ*pbp2b*** | **Moenomycin** | **Depletion** | **Ctr** | **Depletion** | **Ctr** |
| 1 | 345.2 | Linear | Monomer | 4.2 | 5.9 | 4.9 | 4.8 | 4.2 | 4.8 | 4.3 | 4.9 | 4.0 | 4.6 | 4.1 |
| 2 | 487.3 | Linear | Monomer | 1.6 | 1.3 | 1.4 | 7.6 | 1.2 | 1.2 | 2.3 | 1.6 | 1.9 | 1.0 | 2.4 |
| 3 | 503.4 | Branched | Monomer | 18.9 | 18.4 | 18.1 | 15.0 | 20.0 | 21.6 | 18.7 | 20.3 | 18.7 | 17.0 | 18.0 |
| I | 487.4 | Branched | Monomer | 16.7 | 17.0 | 17.3 | 13.8 | 19.2 | 20.3 | 21.1 | 21.6 | 15.6 | 16.6 | 16.0 |
| 4 | 743.4 | Linear | Dimer | 0.3 | 0.7 | 0.4 | 1.5 | 0.4 | 0.4 | 0.5 | 0.5 | 0.6 | 0.9 | 0.7 |
| II | 645.6 | Branched | Monomer | 1.6 | 2.1 | 1.3 | 1.1 | 1.5 | 0.8 | 1.3 | 1.1 | 2.4 | 3.2 | 2.4 |
| III | 629.4 | Branched | Monomer | 1.7 | 2.2 | 1.3 | 1.0 | 1.4 | 0.7 | 1.4 | 1.1 | 2.3 | 3.6 | 2.9 |
| 5 | 901.5 | Branched | Dimer | 6.1 | 6.5 | 6.1 | 6.5 | 5.1 | 4.8 | 7.0 | 6.4 | 5.6 | 7.0 | 6.2 |
| 6a | 885.5 | Branched | Dimer | 2.7 | 3.6 | 3.3 | 3.6 | 2.8 | 3.0 | 2.2 | 3.0 | 2.6 | 2.5 | 2.1 |
| 6b | 901.5 | Branched | Dimer | 0.5 | 0.8 | 0.5 | 0.7 | 0.5 | 0.4 | 0.4 | 0.5 | 1.0 | 0.5 | 0.3 |
| 7 | 1059.6 | Branched | Dimer | 7.5 | 9.1 | 8.8 | 11.9 | 7.4 | 7.6 | 8.5 | 8.8 | 10.2 | 9.3 | 11.1 |
| IV | 1044.3 | Branched | Dimer | 18.1 | 19.0 | 18.6 | 19.2 | 19.1 | 18.3 | 15.8 | 15.1 | 18.4 | 15.2 | 15.8 |
| 8 | 1300.5 | Branched | Trimer | 0 | 0 | 0 | 0 | 0 | 0 | 0 | 0 | 0 | 0 | 0 |
| 9 | 1458.6 | Branched | Trimer | 1.8 | 1.0 | 1.4 | 1.3 | 1.3 | 1.4 | 1.8 | 1.4 | 2.2 | 2.6 | 2.3 |
| V | 1044.3 | Branched | Dimer | 2.6 | 3.0 | 2.0 | 2.5 | 2.1 | 2.4 | 3.6 | 2.1 | 2.8 | 3.5 | 2.7 |
| VI | 1028.1 | Branched | Dimer | 8.7 | 4.9 | 7.0 | 4.6 | 7.0 | 6.6 | 6.6 | 5.8 | 6.8 | 7.3 | 7.5 |
| VII | 1584.9 | Branched | Trimer | 4.1 | 2.6 | 4.3 | 3.0 | 4.2 | 3.3 | 2.2 | 3.3 | 2.6 | 2.2 | 2.4 |
| VIII | 1584.9 | Branched | Trimer | 2.0 | 1.3 | 2.0 | 1.4 | 1.7 | 1.7 | 1.5 | 1.9 | 1.8 | 2.2 | 2.2 |
| IX | 1568.9 | Branched | Trimer | 0.8 | 0.5 | 1.1 | 0.5 | 1.0 | 0.8 | 0.6 | 0.4 | 0.3 | 0.9 | 0.8 |
| **Total** | | | | 100 | 100 | 100 | 100 | 100 | 100 | 100 | 100 | 100 | 100 | 100 |
| **Monomers (%)** | | | | 44.9 | 47.0 | 44.4 | 43.2 | 47.6 | 49.4 | 49.2 | 50.7 | 45.0 | 46.0 | 45.9 |
| **Oligomers (%)** | | | | 55.1 | 53.0 | 55.6 | 56.8 | 52.4 | 50.6 | 50.8 | 49.3 | 55.0 | 54.0 | 54.1 |
| **Linear oligomers (%)** | | | | 0.3 | 0.7 | 0.4 | 1.5 | 0.4 | 0.4 | 0.5 | 0.5 | 0.6 | 0.9 | 0.7 |
| **Branched oligomers (%)** | | | | 54.8 | 52.3 | 55.2 | 55.3 | 52.0 | 50.2 | 50.3 | 48.8 | 54.4 | 53.2 | 53.4 |
| **Linear peptides (%)** | | | | 6.1 | 8.0 | 6.7 | 13.8 | 5.8 | 6.4 | 7.2 | 7.0 | 6.5 | 6.5 | 7.2 |
| **Branched peptides (%)** | | | | 93.9 | 92.0 | 93.3 | 86.2 | 94.2 | 93.6 | 92.8 | 93.0 | 93.5 | 93.5 | 92.8 |
| **B/L peptides** | | | | 15.3 | 11.5 | 14.0 | 6.2 | 16.2 | 14.7 | 13.0 | 13.2 | 14.3 | 14.5 | 12.8 |

Table S3: Stem peptide composition of Pen6 and PBP inhibited or knockout mutants.

^a^Stem peptides were identified based on molecular mass published in previous literature (2-4)

Table S4: Strains and mutants used in this work.

| **Strains** | **Relevant characteristics** | **Reference** |
| --- | --- | --- |
| ***Streptococcus oralis*:** | | |
| Uo5 | A high level β-lactam resistant isolate of *S. oralis* isolated from a Hungarian nasal swab | (6) |
| ***Streptococcus pneumoniae*:** | | |
| RH425 | R6 derivative, but Δ*comA*::*ermAM*, *rpsL1*; Ery^r^ , Sm^r^ | (7) |
| SPH131 | RH425, but ∆*IS1167*::*P1-comR*, ∆*cps*::P*_comX_*-janus; Ery^r^ , Kan^r^ | (5) |
| Pen6 | R6Hex transformant with chromosomal DNA from penicillin-resistant clinical isolate 8249 and selected for Pen^r^ | (8) |
| MH10 | RH425, but *pbp2*x^mos^; Ery^r^ , Sm^r^ | This work |
| MH56 | RH425, but *pbp2x*^mos^*pbp1a*^mos^; Ery^r^ , Sm^r^ | This work |
| MH68 | RH425, but *pbp2x*^mos^*pbp2b*^mos^; Ery^r^ , Sm^r^ | This work |
| MH83 | RH425, but *pbp2x*^mos^*pbp1a*^mos^*pbp2b*^mos^; Ery^r^ , Sm^r^ | This work |
| MH105 | RH425, but ∆*IS1167*::*P1-comR*, ∆*cps*::P*_comX_* -*murM*^Uo5^; Ery^r^ , Sm^r^ | This work |
| MH108 | RH425, but *pbp2x*^mos^*pbp1a*^mos^*pbp2b*^mos^, Δ*murMN*::janus; Ery^r^ , Kan^r^ | This work |
| MH130 | RH425, but *pbp2x*^mos^*pbp1a*^mos^*pbp2b*^mos^, Δ*IS1167*::janus; Ery^r^ , Kan^r^ | This work |
| MH132 | RH425, but *pbp2x*^mos^*pbp1a*^mos^*pbp2b*^mos^, ∆*IS1167*::*P1-comR*; Ery^r^ , Sm^r^ | This work |
| MH134 | RH425, but *pbp2x*^mos^*pbp1a*^mos^*pbp2b*^mos^, ∆*IS1167*::*P1-comR*, ∆*cps*::P*_comX_* -janus; Ery^r^ , Kan^r^ | This work |
| MH136 | RH425, but ∆*IS1167*::*P1-comR*, ∆*cps*::P*_comX_* -*murM*^Uo5^, Δ*murMN*::janus; Ery^r^ , Kan^r^ | This work |
| MH138 | RH425, but *pbp2x*^mos^*pbp1a*^mos^*pbp2b*^mos^, ∆*IS1167*::*P1-comR*, ∆*cps*::P*_comX_* -*murM*^Uo5^; Ery^r^ , Sm^r^ | This work |
| MH141 | RH425, but *pbp2x*^mos^*pbp1a*^mos^*pbp2b*^mos^, ∆*IS1167*::*P1-comR*, ∆*cps*::P*_comX_* -*murM*^Uo5^, Δ*murMN*::janus; Ery^r^ , Kan^r^ | This work |
| MH144 | RH425, but *pbp2x*^mos^*pbp1a*^mos^*pbp2b*^mos^, ∆*IS1167*::*P1-comR*, ∆*cps*::P*_comX_* -*murM*^R6^; Ery^r^ , Sm^r^ | This work |
| MH146 | RH425, but ∆*is1167*::*P1-comR*, ∆*cps*::P*_comX_* -*murM*^R6^, Δ*murMN*::janus; Ery^r^ , Kan^r^ | This work |
| MH147 | RH425, but *pbp2x*^mos^*pbp1a*^mos^*pbp2b*^mos^, ∆*IS1167*::*P1-comR*, ∆*cps*::P*_comX_* -*murM*^R6^, Δ*murMN*::janus; Ery^r^ , Kan^r^ | This work |
| MH149 | RH425, but ∆*IS1167*::*P1-comR*, ∆*cps*::P*_comX_* -*murM*^Uo5^, Δ*murN*::janus; Ery^r^ , Kan^r^ | This work |
| RSG66 | RH425, but Δ*murM*::janus; Ery^r^ , Kan^r^ | This work |
| RSG69 | RH425, but *pbp2x*^mos^*pbp1a*^mos^*pbp2b*^mos^, Δ*murM*::janus; Ery^r^ , Kan^r^ | This work |
| RSG73 | RH425, but *murM^Uo5^*; Ery^r^ , Sm^r^ | This work |
| RSG75 | RH425, but *pbp2x*^mos^*pbp1a*^mos^*pbp2b*^mos^, *murM*^Uo5^; Ery^r^ , Sm^r^ | This work |
| RSG173 | Pen6, but Sm^r^ | This work |
| RSG183 | Pen6, but Δ*murM*::janus; Kan^r^ | This work |
| RSG185 | Pen6, but Δ*eloR*::janus; Kan^r^ | This work |
| RSG186 | Pen6, but *murM*^R6^; Sm^r^ | This work |
| RSG189 | Pen6, but Δ*eloR*::DEL; Sm^r^ | This work |
| RSG192 | Pen6, but Δ*IS1167*::janus;Kan^r^ | This work |
| RSG194 | Pen6, but ∆*IS1167*::*P1-comR*; Sm^r^ | This work |
| RSG199 | Pen6, but ∆*IS1167*::*P1-comR*, ∆*cps*::P*_comX_* -janus; Kan^r^ | This work |
| RSG200 | RH425, but ∆*IS1167*::*P1-comR*, ∆*cps*::P*_comX_* -*murM*^R6^; Sm^r^ | This work |
| RSG203 | Pen6, but ∆*IS1167*::*P1-comR*, ∆*cps*::P*_comX_* -*pbp2a*; Sm^r^ | This work |
| RSG206 | Pen6, but ∆*IS1167*::*P1-comR*, ∆*cps*::P*_comX_* -*pbp2a*, Δ*pbp2a*::janus; Kan^r^ | This work |
| RSG207 | Pen6, but ∆*is1167*::*P1-comR*, ∆*cps*::P*_comX_* -*pbp2a*, Δ*pbp2a*::DEL; Sm^r^ | This work |
| RSG208 | Pen6, but ∆*is1167*::*P1-comR*, ∆*cps*::P*_comX_* -*pbp2a*, Δ*pbp2a*::DEL, Δ*pbp1a*::janus; Kan^r^ | This work |
| RSG214 | Pen6, but Δ*murM*::DEL; Sm^r^ | This work |
| RSG219 | Pen6, but Δ*murM*::DEL, Δ*rsh*::janus; Kan^r^ | This work |
| RSG234 | RH425, but ∆*is1167*::*P1-comR*, ∆*cps*::P*_comX_* -*murM*^R6^, Δ*murM*::janus; Kan^r^ | This work |
| RSG235 | Pen6, but ∆*is1167*::*P1-comR*, ∆*cps*::P*_comX_* -*alaRS*_editing_; Sm^r^ | This work |
| RSG243 | Pen6, but ∆*is1167*::*P1-comR*, ∆*cps*::P*_comX_* -*alaRS*_editing_, Δ*murM*::janus; Kan^r^ | This work |
| RSG399 | RH425, but *pbp2x*^mos^*pbp1a*^mos^*pbp2b*^mos^, *murMN*^Pen6^; Ery^r^ , Sm^r^ | This work |
| JM5 | Pen6, but Δ*pbp1b*::janus; Kan^r^ | This work |
| JM6 | Pen6, but Δ*pbp2a*::janus; Kan^r^ | This work |
| JM9 | Pen6, but Δ*pbp1a*::janus; Kan^r^ | This work |
| JM12 | Pen6, but Δ*eloR*::DEL, Δ*pbp2b*::janus; Kan^r^ | This work |
| AW520 | Pen6, but ∆*is1167*::*P1-comR*, ∆*cps*::P*_comX_* -*pbp2x*^Pen6^; Sm^r^ | This work |
| AW524 | Pen6, but ∆*is1167*::*P1-comR*, ∆*cps*::P*_comX_* -*pbp2x* ^Pen6^, Δ*pbp2x*::janus; Kan^r^ | This work |
| AW594 | Pen6, but ∆*is1167*::*P1-comR*, ∆*cps*::P*_comX_* -*pbp2x* ^Pen6^, Δ*pbp2x*::janus, Δ*lytA*::*aad9*; Sm^r^, Spc^r^ | This work |
| AW627 | Pen6, but *murM^K35A, W38A^*; Sm^r^ | This work |
| AW656 | Pen6, but *flag-murM*; Sm^r^ | This work |
| AW657 | Pen6, but *flag-murM^K35A, W38A^*; Sm^r^ | This work |

Table S5: Primers used in this work

| **Primer** | **Description** | **Sequence(5'-->3')** | **Reference** |
| --- | --- | --- | --- |
| **Primers to amplify the *pbp1a*^Uo5^ amplicon and sequencing of the mutants** | | | |
| MVH26 | ~1000bp upstream *pbp1a*^Uo5^ | CCCTTGTGCTCATATTGTGG | This work |
| MVH27 | ~1000bp downstream *pbp1a*^Uo5^ | TCTGAGCCAACTAATGCCAAC | This work |
| MVH40 | ~500 bp in *pbp1a*^Uo5^ | AGAGATCTTGACCTACTAC | This work |
| MVH41 | ~1000 bp in *pbp1a*^Uo5^ | TCATTGCTCAGTTAGGTTCTCG | This work |
| MVH42 | ~1500 bp in *pbp1a*^Uo5^ | GTATTTAGTGATGGTAGC | This work |
| **Primers to amplify the *pbp2b*^Uo5^ amplicon and sequencing of the mutants** | | | |
| MVH24 | ~1000bp upstream *pbp2b*^Uo5^ | AGGCATAAATCAAATCTATTAAAATG | This work |
| MVH25 | ~1000bp downstream *pbp2b*^Uo5^ | TGATTTTGCTTCTTGCTCGTG | This work |
| MVH37 | ~500 bp in *pbp2b*^Uo5^ | CTATCTCTTTAGCCAGCTCAATG | This work |
| MVH38 | ~1000 bp in *pbp2b*^Uo5^ | CTGAAGGTGTCTATGCAGTAG | This work |
| MVH39 | ~1500 bp in *pbp2b*^Uo5^ | GCCAGTTTGATAACTACACACC | This work |
| **Primers to amplify the *pbp2x*^Uo5^ amplicon and sequencing of the mutants** | | | |
| MVH22 | ~1000bp upstream *pbp2x*^Uo5^ | TGGTGTCCAGGAAATTGATGG | This work |
| MVH23 | ~1000bp downstream *pbp2x*^Uo5^ | TGTAATCAAAAGTTAGTTTTACAG | This work |
| MVH34 | ~500 bp in *pbp2x*^Uo5^ | GATGTCCATTAAACAAGAC | This work |
| MVH35 | ~1000 bp in *pbp2x*^Uo5^ | GGATCAGGCATGAAGGTTATG | This work |
| MVH36 | ~1500 bp in *pbp2x*^Uo5^ | CACATGATCTTAGTTGGGACG | This work |
| **Primers to amplify *murM* and *murN* sequences** | | | |
| VE47 | ~1000bp upstream *murM*^R6^ | ACCAGTAGTCATGGAAGCAAA | (9) |
| KHB199 | ~1000bp downstream *murN*^R6^ | CACAATTTCAGACACCAGAGC | (10) |
| MVH43 | ~100 bp upstream *murM*^R6^ (also ~1300bp upstream *murN*^R6^) | CTTAGTTTGAACTTCAGCATAG | This work |
| MVH44 | ~100 bp downstream *murN*^R6^ (also ~1300bp downstream *murM*^R6^) | GCCAGCGCATGTCTCTCC | This work |
| MVH45 | ~100 bp downstream *murM*^R6^ | CTAGCAAATCCCCCATCTGG | This work |
| **Primers to amplify the Janus cassette** | | | |
| Kan484F | Start Janus cassette | GTTTGATTTTTAATGGATAATGTG | (11) |
| RpsL41R | End Janus cassette | CTTTCCTTATGCTTTTGGAC | (11) |
| **Primers to create the Δ*murMN*::janus amplicon** | | | |
| VE47 | ~1000bp upstream *murM*^R6^ | ACCAGTAGTCATGGAAGCAAA | (9) |
| KHB199 | ~1000bp downstream *murN*^R6^ | CACAATTTCAGACACCAGAGC | (10) |
|  | Template strain: MH110 (Δ*comA*, Δ*murMN*::janus, Ery^r^ Kan^r^) |  | (10) |
| **Primers to create the *ΔmurN*::janus amplicon** | | | |
| MVH43 | ~100 bp upstream *murM*^R6^ (also ~1300bp upstream *murN*^R6^) | CTTAGTTTGAACTTCAGCATAG | This work |
| KHB199 | ~1000bp downstream *murN*^R6^ | CACAATTTCAGACACCAGAGC | (10) |
| KHB198 | End Janus cassette, **overlap just down *murN*^R6^** | CTAAACGTCCAAAAGCATAAGGAAAG**GATGAAAAAGTCAGTATTTAGATT** | (10) |
| MVH49 | start Janus cassette, **overlap start *murN*^R6^** | CACATTATCCATTAAAAATCAAAC*CTTC***TTTCGTGAGTGTTGTTAG** | This work |
| **Template to amplify the Δ*murM*::janus amplicon** | | | |
| VE47 | ~1000bp upstream *murM*^R6^ | ACCAGTAGTCATGGAAGCAAA | (9) |
| MVH44 | ~100 bp downstream *murN*^R6^ (also ~1300bp downstream *murM*^R6^) | GCCAGCGCATGTCTCTCC | This work |
|  | Template strain: SPH181 (Δ*comA* *P1*::*PcomR*::*comR* *PcomX*::*pbp2b* Δ*pbp2b*^Wt^ Δ*lytA*::Spc^r^ Δ*murM*::Janus Ery^r^ Spc^r^ Kan^r^) | | (9) |
| **Primers to replace murMNR6 with murMUo5** | | | |
| GS428 | just downstream *murN*^R6^ | GATGAAAAAGTCAGTATTTAGATT | This work |
| GS429 | just upstream *murM*^R6^ | TTCCTACTCTCTTTCCTCCA | This work |
| GS430 | just upstream *murM*^R6^, **overlap start *murM*^Uo5^** | TGGAGGAAAGAGAGTAGGAA**ATGTTTACGTATAAAATGAATGTTG** | This work |
| GS431 | just downstream *murN*^R6^, **overlap end *murM*^Uo5^** | AATCTAAATACTGACTTTTTCATC**CTAATTCCTACTTCGAAGTTTC** | This work |
| **Primers to amplify the ∆*is1167*::janus and ∆*is1167*::*P1-comR* amplicons** | | | |
| AmiF | ~1000bp upstream *P1-comR* | CGGTGAAGGAAGTAAGAAGTTT | (7) |
| TreR | ~1000bp downstream *P1-comR* | GTGACGGCAGTCACATTCTC | (7) |
|  | Template strain: RH426 (RH425, but Δ*IS1167*::Janus; Ery^r^ Kan^r^) |  | (7) |
|  | Template strain: SPH131 (RH425, but ∆*is1167*::*P1-comR*, ∆*cps*::*PcomX*::janus) |  | (5) |
| **Primers to amplify the *PcomX*::janus amplicon and create replacements** | | | |
| KHB31 | ~800bp upstream *PcomX* | ATAACAAATCCAGTAGCTTTGG | (5) |
| KHB34 | ~800bp downstream *PcomX*::janus | CATCGGAACCTATACTCTTTTAG | (5) |
| KHB33 | just down *PcomX*::janus | TTTCTAATATGTAACTCTTCCCAAT | (5) |
| KHB36 | end of *PcomX* | TGAACCTCCAATAATAAATATAAAT | (5) |
|  | Template strain: SPH131 (RH425, but ∆*is1167*::*P1-comR*, ∆*cps*::*PcomX*::janus) |  |  |
| **Primers to create the *PcomX*::*murM*^Uo5^ amplicon** | | | |
| KHB31 | ~800bp upstream *PcomX* | ATAACAAATCCAGTAGCTTTGG | (5) |
| KHB34 | ~800bp downstream *PcomX*::janus | CATCGGAACCTATACTCTTTTAG | (5) |
| MVH46 | end of *PcomX*, **overlap start *murM*^Uo5^** | ATTTATATTTATTATTGGAGGTTCA**ATGTTTACGTATAAAATGAATGTTG** | This work |
| MVH47 | just downstream *PcomX*::janus, **overlap end *murM*^Uo5^** | ATTGGGAAGAGTTACATATTAGAAA**CTAATTCCTACTTCGAAGTTTC** | This work |
| **Primers to create the *PcomX*::*murM*^R6^ amplicon** | | | |
| KHB31 | ~800bp upstream *PcomX* | ATAACAAATCCAGTAGCTTTGG | (5) |
| KHB34 | ~800bp downstream *PcomX*::janus | CATCGGAACCTATACTCTTTTAG | (5) |
| KHB374 | end of *PcomX*, **overlap start *murM*^R6^** | ATTTATATTTATTATTGGAGGTTC**AATGTACCGTTATCAAATTGGCAT** | This work |
| KHB375 | just downstream *PcomX*::janus, **overlap end *murM*^R6^** | ATTGGGAAGAGTTACATATTAGAAA**TTACTTTCTATGTTTTTTTCTTAATG** | This work |
| **Primers to create the *murM*::DEL amplicon** | | | |
| RSG46 | Just downstream *murM*^R6^, **overlapp just up *murM*^R6^** | GAGTGTTGTTAGTGCCATATAC**TTCCTACTCTCTTTCCTCCAG** | This work |
| RSG60 | Just downstream *murM*^R6^ | GTATATGGCACTAACAACACTC | This work |
| **Primers to amplify the Δ*eloR*::janus and create the Δ*eloR*::DEL amplicon** | | | |
| DS374 | ~900bp upstream *eloR* | CGAAACCTTGGGATACGCAG | (12) |
| DS377 | ~1000bp downstream *eloR* | CAGCACCCACGTTAAGCAAC | (12) |
| DS390 | Just downtream *eloR***, overlap just up *eloR*** | GAAATAAATAAGGAGGAATCTGGTA**GTAAAATCAGGTTTATCCTGATTTTTTGCTAG** | This work |
| DS378 | Just up *eloR* | TACCAGATTCCTCCTTATTTATTTC | This work |
|  | Template strain: SPH472 (Δ*comA*, Δ*eloR*::janus, m(sf)GFP-*mltG*; Ery^r^, Kan^r^) |  | (12) |
| **Primers to amplify the Δ*pbp2b*::janus amplicon** | | | |
| KHB129 | ~900bp upstream *pbp2b*^R6^ | CGATAAAGAAGAGCATAGGAAG | (9) |
| KHB132 | ~1000bp downstream *pbp2b*^R6^ | TCCCAATCAATGGTTTCATTGG | (9) |
|  | Template strain: SPH156 (Δ*pbp2b*::janus, *PcomX*::*pbp2b*, Kan^r^) |  | (9) |
| **Primers to amplify the Δ*pbp1a*::janus amplicon** | | | |
| MTS5F | ~1000bp upstream *pbp1a*^R6^ | CCTTGTGTTCATAGCGAGG | (13) |
| MTS8R | ~1000bp downstream *pbp1a*^R6^ | AAAACGGCTTTGGTAGCAGATG | (13) |
|  | Template strain: SPH344 (Δ*comA*, *ssbB*::*luc*, Δ*pbp1a*::Janus, Ery^r^, Cm^r^, Kan^r^) |  | (13) |
| **Primers to amplify the Δ*pbp1b*::janus amplicon** | | | |
| MTS9F | ~1200bp upstream *pbp1b*^R6^ | GCCTGTACTTGGTAGTTTGG | (13) |
| MTS12R | ~1000bp downstream *pbp1b*^R6^ | GACTATTCCAGTATAGCAC | (13) |
|  | Template strain: SPH345 (Δ*comA*, *ssbB*::*luc*, Δ*pbp1b*::Janus, Ery^r^, Cm^r^, Kan^r^) |  | (13) |
| **Primers to amplify the Δ*pbp2a*::janus amplicon** | | | |
| MTS1F | ~1000bp upstream *pbp2a*^R6^ | GCACAACTTGTTCGTACTCTTG | (13) |
| MTS4R | ~1000bp downstream *pbp2a*^R6^ | AGGTTTACTTCTGCAACTGTG | (13) |
|  | Template strain: SPH346 (Δ*comA*, *ssbB*::*luc*, Δ*pbp2a*::Janus, Ery^r^, Cm^r^, Kan^r^) |  | (13) |
| **template to amplify the *PcomX*::*pbp2a* amplicon** | | | |
| KHB31 | ~800bp upstream *PcomX* | ATAACAAATCCAGTAGCTTTGG | (5) |
| KHB34 | ~800bp downstream *PcomX*::janus | CATCGGAACCTATACTCTTTTAG | (5) |
| MTS17F | end of *PcomX*, **overlap start *pbp2a*** | ATTTATATTTATTATTGGAGGTTCA**ATGAAATTAGATAAATTATTTGAGAA** | This work |
| MTS18 | just downstream *PcomX*::janus, **overlap end *pbp2a*** | GGGAAGAGTTACATATTAGAAA**TTAGCGAAATAGATTGACTATCG** | This work |
| **Primers to create the Δ*pbp2a*::DEL amplicon** | | | |
| MTS1F | ~1000bp upstream *pbp2a*^R6^ | GCACAACTTGTTCGTACTCTTG | (13) |
| MTS4R | ~1000bp downstream *pbp2a*^R6^ | AGGTTTACTTCTGCAACTGTG | (13) |
| MTS15R | Just downstream *pbp2a*, **overlapp just up *pbp2a*** | GCTAGGCTTTGACAAGCATC**GCGTTTATTTTATCATCTTCATC** | This work |
| MTS16F | Just upstream *pbp2a*, **overlapp just down *pbp2a*** | GATGAAGATGATAAAATAAACGC**GATGCTTGTCAAAGCCTAGC** | This work |
| **Primers to amplify the *rpsL* gene to generate a Sm^r^ resistant strain** | | | |
| DS827 | ~1000bp upstream *rpsL* | CATCTAGGTAATAGCCGTAGTC | This work |
| DS828 | ~1000bp downstream *rpsL* | GGCATCGACGTGAGCCATG | This work |
|  | Template strain: RH425 (R6 derivative, but Δ*comA*::*ermAM*, *rpsL1*; Ery^r^ , Sm^r^) |  | (7) |
| **Primers to create the Δ*pbp2x*::janus amplicon** | | | |
| KHB104 | ~700bp upstream *pbp2x*^R6^ | GAAGTGAAGCCGATTGAGAC | (9) |
| KHB107 | ~700bp downstream *pbp2x*^R6^ | ACACAATTCCGATAATCAAGAG | (9) |
| AW348 | ~100bp upstream *pbp2x*^Pen6^ | TTGGCACCCTATATCGAAAAAG | This work |
| AW349 | Start Janus cassette, **overlap just up *pbp2x*^Pen6^** | CACATTATCCATTAAAAATCAAAC**TCCGCTATTCGAATATTTTCATTG** | This work |
| AW350 | End Janus cassette, **overlap just down *pbp2x*^Pen6^** | GTCCAAAAGCATAAGGAAAG**ATGTTTATTTCCATCAGTGCTGG** | This work |
| AW351 | ~100bp downstream *pbp2x*^Pen6^ | TACTATATTTTGAGCAGCCTAAAG | This work |
| **Primers to create the *PcomX*::*pbp2x*^Pen6^ amplicon** | | | |
| AW354 | end of *PcomX*, overlap start *pbp2x*^Pen6^ | ATTTATATTTATTATTGGAGGTTCA**ATGAAGTGGACAAAAAGAATAACC** | This work |
| AW355 | Just downstream *PcomX*, overlap end *pbp2x*^Pen6^ | ATTGGGAAGAGTTACATATTAGAAA**CAGCACTGATGGAAATAAACATATTA** | This work |
| AW356 | Sequencing primer ~700 bp into *pbp2x*^Pen6^ | CGTCTGGGTAATATTGTCCC | This work |
| **Primers to create the Δ*rsh*::janus amplicon** | | | |
| RSG63 | ~1000bp upstream *rsh* | ACAGGATTCACGGTTTTATGG | This work |
| RSG64 | ~1000bp downstream *rsh* | CGTGCAGGATAGGATACCC | This work |
| RSG61 | End Janus cassette, **overlap just down *rsh*** | GTCCAAAAGCATAAGGAAAG**TTGTCCTAGCTCTTACTAGAAAG** | This work |
| RSG62 | Start Janus cassette, **overlap just up of *rsh*** | CACATTATCCATTAAAAATCAAAC**CTCTACTCTCCAATTCTTCCT** | This work |
| **Primers to create the *PcomX*::*alaRS*_editing_ amplicon** | | | |
| KHB31 | ~800bp upstream *PcomX* | ATAACAAATCCAGTAGCTTTGG | (5) |
| KHB34 | ~800bp downstream *PcomX*::janus | CATCGGAACCTATACTCTTTTAG | (5) |
| RSG65 | end of PcomX, **overlap start *alaRS*_editing_** | ATTTATATTTATTATTGGAGGTTCA**GCGTCAGCTGTCAAGGGTG** | This work |
| RSG66 | just downstream *PcomX*::janus, **overlapp end *alaRS*_editing_** | ATTGGGAAGAGTTACATATTAGAAA**TTACAATTTACCTGCTACTGCATC** | This work |
| **Primers to introduce *murM^K35A,W38A^* and Flag-tag** | |  |  |
| VE47 | ~1000bp upstream *murM*^R6^ | ACCAGTAGTCATGGAAGCAAA | (9) |
| AW496 | Introduces the mutations K35A and W38A in MurM | **CGCATCAGAAGC**CACTTTTTCCCAAGCACTGC | This work |
| AW497 | Introduces the mutations K35A and W38A in MurM | **GAAAAAGTGGCTTCTGATGCG**AATCATGAGAGACTTGGTGTCTA | This work |
| AW509 | Primer used to introduce the flag-tag N-terminally to MurM from Pen6 | ATGGATTATAAAGATCATGATGGTGATTATAAAGATCATGATATTGATTATAAAGATGATGATGATAAATACCGTTATCAAATTGGCATTCC | This work |
| AW510 | Primer used to introduce the flag-tag N-terminally to MurM from Pen6 | CACCATCATGATCTTTATAATCCAT TTCCTACTCTCTTTCCTCCAGT | This work |
| MVH44 | ~100 bp downstream *murN*^R6^ (also ~1300bp downstream *murM*^R6^) | GCCAGCGCATGTCTCTCC | This work |

# References:

1. Filipe SR, Pinho MG, Tomasz A. 2000a. Characterization of the *murMN* operon involved in the synthesis of branched peptidoglycan peptides in *Streptococcus pneumoniae*. Journal of Biological Chemistry 275:27768-27774.

2. Garcia-Bustos J, Chait BT, Tomasz A. 1987. Structure of the peptide network of pneumococcal peptidoglycan. Journal of Biological Chemistry 262:15400-15405.

3. Garcia-Bustos JF, Chait BT, Tomasz A. 1988. Altered peptidoglycan structure in a pneumococcal transformant resistant to penicillin. J Bacteriol 170:2143-7.

4. Severin A, Tomasz A. 1996. Naturally occurring peptidoglycan variants of *Streptococcus pneumoniae*. J Bacteriol 178:168-74.

5. Berg KH, Biørnstad TJ, Straume D, Håvarstein LS. 2011. Peptide-regulated gene depletion system developed for use in *Streptococcus pneumoniae*. J Bacteriol 193:5207-15.

6. Reichmann P, König A, Linares J, Alcaide F, Tenover FC, L, Swidsinski S, Hakenbeck R. 1997. A Global Gene Pool for High-Level Cephalosporin Resistance in Commensal *Streptococcus* Species and *Streptococcus Pneumoniae*. The Journal of Infectious Diseases 176:1001-1012.

7. Johnsborg O, Håvarstein LS. 2009. Pneumococcal LytR, a Protein from the LytR-CpsA-Psr Family, Is Essential for Normal Septum Formation in *Streptococcus pneumoniae*. Journal of Bacteriology 191:5859-5864.

8. Zighelboim S, Tomasz A. 1980. Penicillin-binding proteins of multiply antibiotic-resistant South African strains of *Streptococcus pneumoniae*. Antimicrobial Agents and Chemotherapy 17:434-442.

9. Berg KH, Stamsås GA, Straume D, Håvarstein LS. 2013. Effects of Low PBP2b Levels on Cell Morphology and Peptidoglycan Composition in *Streptococcus pneumoniae* R6. Journal of Bacteriology 195:4342-4354.

10. Straume D, Piechowiak KW, Olsen S, Stamsås GA, Berg KH, Kjos M, Heggenhougen MV, Alcorlo M, Hermoso JA, Håvarstein LS. 2020. Class A PBPs have a distinct and unique role in the construction of the pneumococcal cell wall. Proceedings of the National Academy of Sciences 117:6129-6138.

11. Johnsborg O, Eldholm V, Bjørnstad ML, Håvarstein LS. 2008. A predatory mechanism dramatically increases the efficiency of lateral gene transfer in *Streptococcus pneumoniae* and related commensal species. Mol Microbiol 69:245-53.

12. Stamsås GA, Straume D, Ruud Winther A, Kjos M, Frantzen CA, Håvarstein LS. 2017. Identification of EloR (Spr1851) as a regulator of cell elongation in *Streptococcus pneumoniae*. Molecular microbiology 105:954-967.

13. Straume D, Stamsås GA, Berg KH, Salehian Z, Håvarstein LS. 2017. Identification of pneumococcal proteins that are functionally linked to penicillin‐binding protein 2b (PBP2b). Molecular microbiology 103:99-116.
